# All-Plastic Organic Lasers with Top-Layer Polymeric Resonators: Tunable Emission through Bending and Application to Refractive Index Sensing

**DOI:** 10.1021/acsaelm.5c01592

**Published:** 2025-10-10

**Authors:** Pablo Pasqués-Gramage, Gema Calvillo-Solís, Pedro G. Boj, José A. Quintana, José M. Villalvilla, María A. Díaz-García

**Affiliations:** † Dpto. Física Aplicada and Instituto Universitario de Materiales de Alicante (IUMA), 16718 Universidad de Alicante, Alicante 03080, Spain; ‡ Dpto. Óptica, Farmacología y Anatomía and IUMA, 16718 Universidad de Alicante, Alicante 03080, Spain

**Keywords:** organic distributed feedback laser, all-plastic, plastic substrates, tunable emission, flexible optics, cellulose acetate

## Abstract

All-plastic thin-film organic lasers, in which all the layers comprising the device (active medium, resonator and substrate) are of polymeric nature, are very interesting because they offer the possibility to tune the emission laser wavelength through mechanical deformation (bending). Here, we report all-plastic distributed feedback (DFB) lasers based on top-layer dichromated gelatin resonators (one-dimensional gratings), active layers of polystyrene doped with perylene orange and substrates of cellulose acetate (CA), showing a successful laser performance, comparable to devices based on fused silica substrates. Remarkably, the emission wavelength of the prepared lasers can be tuned by approximately 10 nm through mechanical deformation (bending) thanks to the polymeric nature of all the layers involved in the device. This tuning is primarily due to changes in the grating period, while the layer thickness remains constant. The potential of the devices for refractive index sensing is also demonstrated. These findings highlight the potential of flexible top-layer resonator DFB lasers for applications requiring adjustable emission wavelengths, such as portable or adaptive optical systems. Additionally, the use of CA substrates and simple fabrication processes make these devices cost effective and easy to produce, opening the door to scalable and low-cost production for various optical applications.

## Graphical abstract

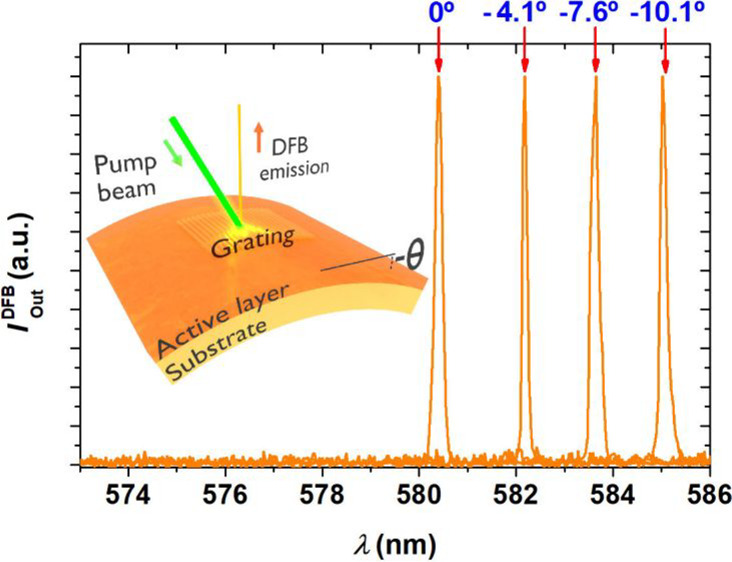

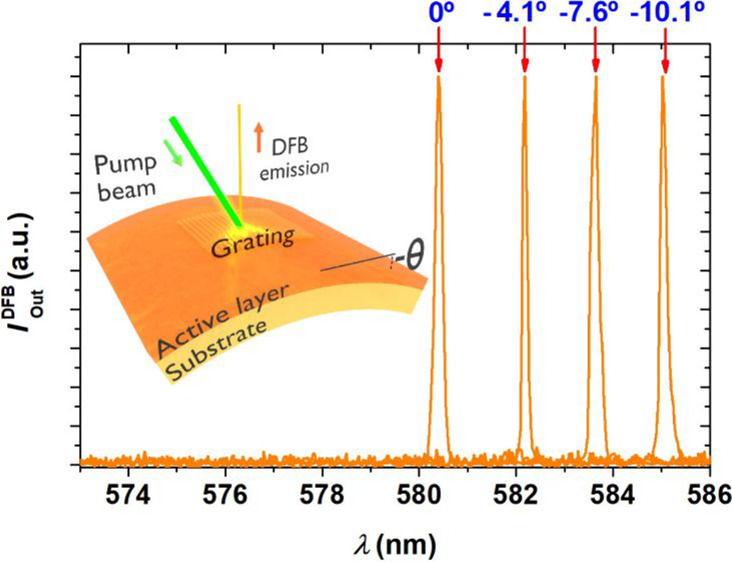

## 1 Introduction

In recent years, the development of flexible photonic devices has garnered considerable attention due to their exceptional potential across a wide range of applications, including sensors, telecommunications, and wearable systems.^1–3^ Within this field, organic distributed feedback (DFB) lasers have distinguished themselves by providing coherent laser emissions with tunable wavelengths, making them highly adaptable for various technological applications.^4^ However, developing completely mechanically flexible devices (including the substrate) remains a challenging task,^5^ especially when trying to maintain optimal performance, efficiency, and stability under changing and deformable conditions.

Conventional organic DFB lasers are typically fabricated on inorganic rigid substrates (for example, fused silica, FS) with engraved relief gratings, which offer high stability and control over the optical properties of the grating.^4^ Indeed, these have been generally the devices that have shown the best performance in terms of threshold (minimum excitation energy needed to operate). In recent years, there have been numerous efforts in developing lasers with solution-processed resonators in the pursuit of all-plastic devices,^6–8^ although generally their performance has been inferior in comparison to systems with inorganic gratings. In this context, devices based on top-layer resonators of dichromated gelatin (DCG) have shown great potential toward this end because their threshold performance is similar to that of systems with the same active material and resonator characteristics, but with standard gratings,^9,10^ while offering advantages in terms of laser efficiency. Indeed, these top-layer DCG-based devices have been successfully applied to a variety of active compounds emitting from the blue to the infrared.^11–13^ Moreover, an advantage of the top-resonator geometry is that it enables multicolour emission devices by engraving gratings with different periods in the same device, thanks to the use of holographic lithography (HL).

Another step needed to obtain an all-plastic device is the inclusion of a polymeric flexible substrate. This would revolutionize the design and functionality of these devices while also reducing production costs and improving integration into portable systems.^14^ There have been some efforts toward obtaining all-plastic lasers, such as those incorporating encapsulated star-shaped oligofluorene gain media,^15^ conjugated polymer-based DFB lasers emitting in the blue-green spectral region,^16^ devices including volume gratings,^17^ or flexible and ultralightweight polymer membrane lasers that have demonstrated the effectiveness of flexible materials for DFB devices in portable applications.^18^ A particular material that has shown good potential as a substrate for organic DFB lasers is cellulose acetate (CA), thanks to its flexibility, transparency, and ease of processing.^19^ Flexible DFB lasers using nanoimprinted cellulose diacetate have been demonstrated, highlighting the potential for flexible substrates in photonic device integration.^20^ The use of a flexible substrate is of particular interest because the optical behavior of the device and the wavelength can potentially be tuned through substrate deformation.^21–23^ In this context, CA offers a unique combination of optical properties, such as high transparency and a refractive index close to that of FS, alongside mechanical flexibility and compatibility with scalable fabrication techniques. These characteristics make CA especially highly suitable for flexible DFB laser applications, in contrast to other flexible materials that may not meet the stringent optical or processing requirements of the device.

In the present work, we focus on the fabrication and characterization of organic DFB devices with top-layer DCG resonators using CA as substrates and polystyrene (PS) doped with perylene orange (PDI-O) as the active medium. To date, all reported top-layer DCG resonator DFB lasers have used FS as the substrate,^9–13^ so the CA-based devices prepared here are compared to similar ones based on FS (instead of CA). We have first optimized the grating fabrication procedure for the CA-based lasers, which has been particularly challenging in the holography exposure step, which is very sensitive to vibrations and lack of rigidity of the sample. An aspect of interest here is to explore the possibility of tuning the laser emission wavelength through deformation (bending) of the device. This ability to adjust wavelengths is particularly valuable for applications requiring adaptability to varying external conditions, such as strain sensors or environmental monitoring devices. Moreover, we have investigated the feasibility of the prepared CA-based DFB devices as refractive index sensors, comparing their performance to devices fabricated on FS, which have already demonstrated effectiveness for such purposes.^24^ By exploring the tuning of the laser emission wavelength through device deformation (bending), this study seeks to provide a deeper understanding of the opportunities and challenges associated with top-layer DCG resonator DFB flexible devices based on CA substrates. The results of this research are expected to open new possibilities for integrating these devices into adaptable and low-cost optical sensor systems.

## 2 Results and Discussion

### 2.1 ASE Characterization and Comparison

We first characterize the optical properties of the active material (PS films with 1 wt % of PDI-O dispersed in them), deposited over CA and FS substrates, without DCG resonators. Their absorption, photoluminescence (PL), and amplified spontaneous emission (ASE) spectra are shown in Figure 1a. The spectra clearly exhibit similarities in spectral shape and emission wavelengths, suggesting that the active film behaves consistently on both substrates, with absorption peak wavelengths at 459, 490, and 527 nm and PL peaks at 537 and 578 nm. This indicates that the interaction between PDI-O and the substrate does not significantly affect the optical properties, which are almost identical for both substrates.

**Figure 1:**
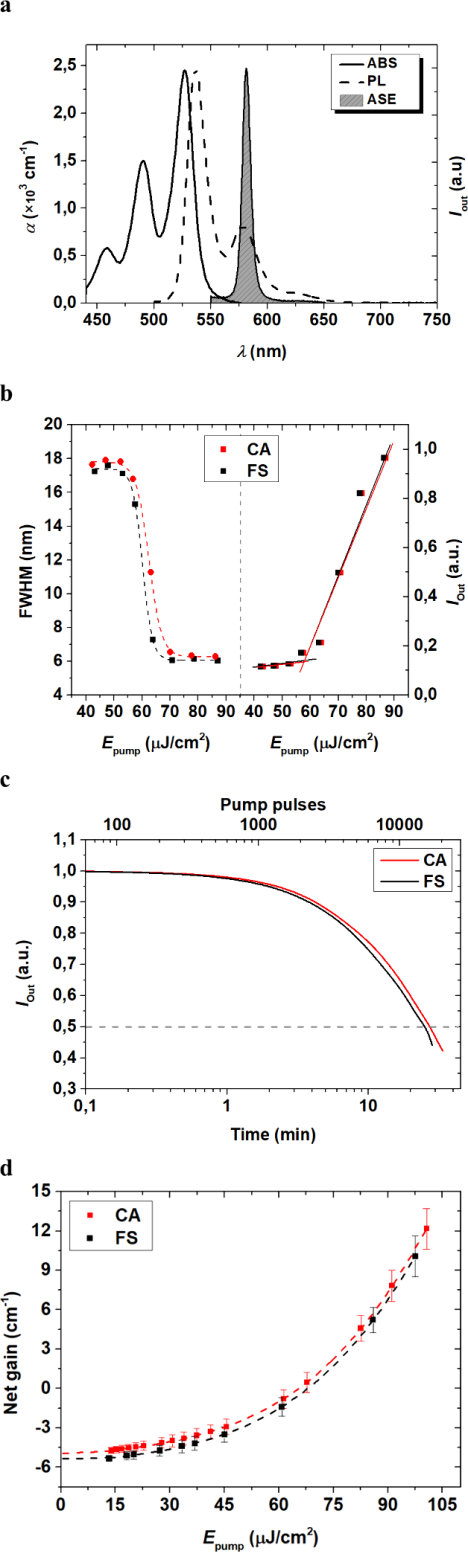
Comparison of ASE properties of PDI-O doped films on FS and CA substrates. (a) Absorption (left axis), emission (right axis, “a.u.”: arbitrary units) and ASE spectra (right axis) of PDI-O doped PS films (1 wt %) under pulsed excitation (λ_p_ = 532 nm) on CA. (b) Output intensity and emission line width (FWHM) versus pump energy density for films on FS (black squares) and CA (red squares) at λ_p_ = 532 nm. (c) ASE intensity (*I*_Out_) versus time and number of pump pulses for 1 wt % PDI-O on FS and CA. Lifetime (τ_1/2_^ASE^) is when *I*_Out_ decays to half. (d) Net gain versus pump energy density at ASE peak for FS and CA substrates. Full lines are guides to the eye.

All relevant ASE performance parameters are collected in Table 1: ASE line width (defined as the full width at half-maximum, FWHM_ASE_, under excitation above threshold), excitation ASE threshold and ASE photostability (ASE operational lifetime, τ_1/2_^ASE^). The ASE line width is nearly identical for both substrates. Figure 1b shows the energy thresholds required to activate ASE in both samples (with FS and CA) showing similar values for both substrates: 62 μJ cm^–2^ and 60 μJ cm^–2^ for CA and FS, respectively. Regarding photostability (see Figure 1c), it is seen that τ_1/2_^ASE^ under extreme pumping conditions (2500 kW cm^–2^, which is around 200 times over the threshold) is slightly longer for the CA-based sample (τ_1/2_^ASE^ = 1.7 × 10^4^ pump pulses) compared to the one based on FS (τ_1/2_^ASE^ = 1.5 × 10^4^ pump pulses), although this is not a significant difference. It should be noted that these values are obtained under extreme pumping conditions. Longer stabilities are obtained if pumping at moderate conditions (twice times over threshold) as reported elsewhere.^9^ The active PS layer doped with PDI-O (1 wt %) is responsible for the absorption, emission, and ASE properties, and since its composition and thickness are the same in both cases, the optical and ASE characteristics primarily depend on the interaction of light with the active layer, rather than the substrate properties. The net gain is also similar for both (Figure 1d), indicating that signal amplification is not significantly influenced by the substrate. The results show that the optical and ASE properties of PDI-O doped PS films are primarily determined by the active layer and not by the substrate differences, as both provide efficient conditions for activating the film.

**Table 1:** Optical and ASE Properties of PS Films Doped with 1 wt % of PDI-O over Different Substrates (CA and FS)

Substrate	*h* ^a^ [nm]	α[λ_p_]^b^ [cm^–1^]	λ_ASE_ ^c^ [nm]	FWHM_ASE_ ^d^ [nm]	*E*_th–ASE_ ^e^ [μJ cm^–2^]	*I*_th–ASE_ ^e^ [kW cm^–2^]	τ_1/2_^ASE,^ ^f^ [pp] (2500 kW cm^–2^)
CA	515	2.4 × 10^3^	581	6.3	62	15	1.7 × 10^4^
FS	525	2.3 × 10^3^	581	6.1	60	15	1.5 × 10^4^

a Film thickness (error ≈ 2%).

b Absorption coefficient at the pump wavelength λ_p_ = 532 nm (error ≈ 2%).

c ASE wavelength (error is ± 0.5 nm).

d ASE line width (error is ± 0.8 nm), defined as the full width at half-maximum, FWHM, well above the threshold.

e ASE threshold (error ≈ 20%).

f ASE operational lifetime, characterized by the photostability half-life τ_1/2_^ASE^ (error ≈ 20%). Parameter expressed in pump pulses (pp). The pump intensity is indicated in brackets and correspond to extreme pump conditions: 2 orders of magnitude over threshold.

### 2.2 DFB Laser Fabrication and Characterization

Laser devices with samples such as those studied in section 2.1, were prepared by depositing DCG layers on top, and engraving one-dimensional surface-relief gratings (by HL and subsequent dry etching) that serve as the laser resonator (see device structure in Figure 2a). This grating serves two essential functions in the laser’s operation: it provides feedback for the light propagating along the layer; and besides, it enables light extraction from the device. Regarding the first function, the wavelength that is enhanced as it propagates along the layer is the Bragg wavelength, λ_Bragg_,^4,25^ which is defined by the following equation:

1 $$m\lambda_{Bragg}=2n_{eff}\Lambda$$

where *m* is the operating order (*m* = 2 for all the devices in this work), *n*_eff_ is the effective refractive index of the waveguide, which depends on the active film thickness *h*, as well as the refractive index of the active layer, substrate, and cover. The second function (light extraction) corresponds to first-order diffraction, which for the prepared devices occurs preferentially in a direction perpendicular to the sample surface at a wavelength λ_DFB_ close to λ_Bragg_. The geometric parameters of the grating have been carefully adjusted to achieve optimal laser performance. In particular, the modulation depth of the grating, *d*, has been chosen to be approximately 110 nm to achieve a low threshold while maintaining reasonable laser efficiency, according to previous studies.^10^ The grating period value, Λ, has been adjusted through the HL fabrication step to produce laser emission close to the ASE wavelength (λ_ASE_), where the gain is maximum.

**Figure 2:**
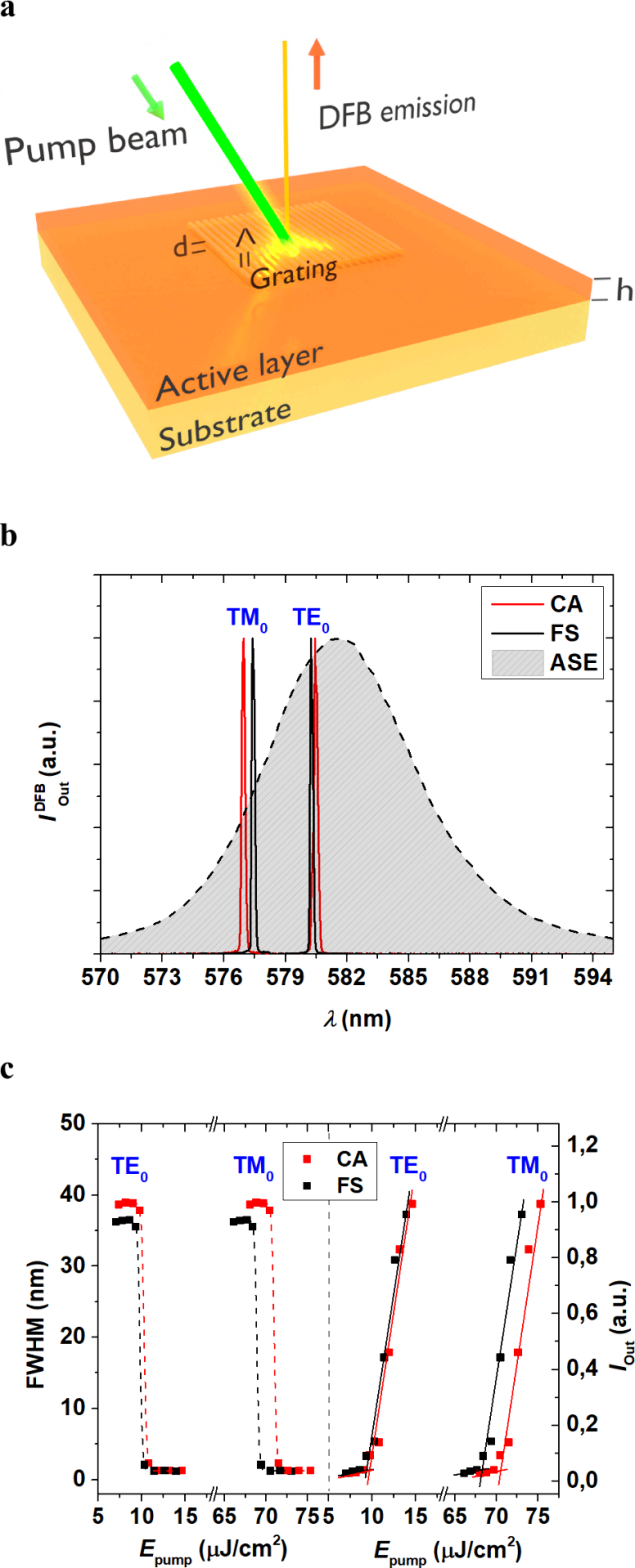
Device architecture and optical performance of 1/FS and 1/CA DFB lasers based on PDI-O doped films. (a) Device layout, featuring a top-layer resonator made up of a relief grating (Λ, grating period; *d*, grating depth) positioned on the active film (*h*, film thickness). (b) DFB laser spectra for devices based on films containing 1 wt % of PDI-O, using FS substrate (black lines) and CA substrate (red lines). (c) Output intensity (right axis) and emission line width, defined as the full width at half-maximum, FWHM (left axis) as a function of the pump energy density, for the same film under λ_p_ = 532 nm with substrates of FS (black squares) and CA (red squares), with *d* ≈ 110 nm.

The value of *h* has also been adjusted, for each type of substrate, to allow the active films to support only the TE_0_ and TM_0_ waveguide modes with high confinement.^26^ This means that *h* must be greater than the calculated cutoff thickness (*h*_cutoff_) for the propagation of such modes, and lower than that of higher-order modes (see Figure S1 and Table S1 in the Supporting Information).

Two DFB laser devices were fabricated for each substrate type: one with a grating depth of around 110 nm and the other with a grating depth of around 50 nm (devices labeled as 1/CA and 1/FS; and as 2/CA and 2/FS, respectively, see Table 2).

**Table 2:** Properties of Top-Layer DCG Resonator DFB Lasers Based PDI-O-Doped PS Films with Different Substrates (CA and FS)

Device Tag/Subs.	Mode	Polarizat. Orient.^a^	*h* ^b^ [nm]	*d* ^c^ [nm]	Λ^d^ [nm]	λ_*DFB*_ ^e^ [nm]	FWHM_DFB_ ^f^ [nm]	*E*_th‑DFB_ ^g^ [μJ cm^–2^]	*I*_th‑DFB_ ^g^ [kW cm^–2^]
1/CA	TE_0_	Parallel	518	113	372.2	580.47	0.08	10	1.0
	TM_0_	Perpend.	518	113	372.2	577.05	0.11	71	7.1
1/FS	TE_0_	Parallel	525	111	371.9	580.43	0.09	10	1.0
	TM_0_	Perpend.	525	111	371.9	577.41	0.11	69	6.9
2/CA	TE_0_	Parallel	521	51	372.1	580.26	0.09	11	1.1
2/FS	TE_0_	Parallel	530	49	371.7	580.33	0.09	10	1.1

a Orientation of the diffraction grating lines with respect to the laser polarization: “Parallel” indicates that the grating lines are aligned parallel to the laser polarization direction; “Perpend.” indicates they are orthogonal.

b Film thickness (error ≈ 2%).

c Grating depth (error is ± 5 nm).

d Grating period (error is ± 0.5 nm).

e DFB wavelength (error is ± 0.07 nm).

f DFB line width (error is ± 0.1 nm), defined as the full width at half-maximum, FWHM, well above the threshold.

g DFB threshold (error ≈ 10%).

In the device layout, shown in Figure 2a, the top-layer resonator is composed of a relief grating with a specific period (Λ) and depth (*d*). This resonator is positioned on top of the active film with a thickness of *h*. The relief grating structure plays a crucial role in the device’s resonant behavior, influencing the laser’s emission characteristics. The grating profile is illustrated through SEM and FESEM images for a DCG grating with a period of Λ = 371.7 nm and a depth of about *d* ≈ 110 nm in Figure S2 in the Supporting Information.^10^ The interaction between the grating and the active material determines the wavelength and other optical properties of the DFB laser. The DFB laser spectra, shown in Figure 2b, demonstrate that devices made with FS (black lines) and CA (red lines) exhibit similar emissions for peaks associated with both TE_0_ and TM_0_ waveguide modes. In both cases, the active material (PDI-O dispersed in PS) and the resonant layer are the dominant factors determining the emission wavelength. The emission lines are virtually identical, suggesting that the optical emission characteristics do not depend on the substrate.

The spatial reproducibility of the CA gratings was assessed by recording multiple DFB spectra across a single device, where the resonator was deposited onto the active film. This was achieved by scanning the excitation beam across the grating area of 1 × 1 cm^2^. An example of the resulting emission maps is provided in Figure S3 in the Supporting Information. Particular focus was placed on evaluating spectral quality and peak position, with the maps demonstrating consistent performance throughout the entire sample.

The threshold performance of the prepared devices appears to be independent of the type of substrate, as illustrated in Figure 2c for devices 1/CA and 1/FS. This figure shows the output intensity and the emission line width (FWHM) as a function of pump energy density (at λ_p_ = 532 nm). Devices based on FS and CA show nearly identical responses, with excitation thresholds for devices 1/CA and 1/FS of around 70 μJ cm^–2^, for the peaks associated with the TM_0_ mode, and of around 10 μJ cm^–2^ for the peaks associated with the TE_0_ mode, see Table 2. These results indicate minimal differences between the two substrates, even regarding emission efficiency and line width. This behavior is also seen in devices with a grating depth of around 50 nm (2/CA and 2/FS), see Table 2.

Regarding the photostability of the DFB lasers, previous studies with similar devices but prepared over FS substrates, indicate that it is comparable to that of films without resonators (ASE photostabilities) and independent of device geometry.^11^ To confirm this for the CA-based DFBs developed in this work, operational lifetime measurements were performed. As expected, similar DFB half-lives of approximately 1.5 × 10^4^ pump pulses (under pumping at 2500 kW cm^–2^) were measured for both CA and FS substrates, in accordance with the ASE photostability values. Additionally, all devices exhibited excellent long-term stability under dark storage, maintaining their threshold and photostability performance even after several months.

Despite the inherent differences between FS and CA substrates, such as their optical absorption (see Figure S4), refractive indices (see Figure S5 and eqs S1–S3),^27^ and mechanical stiffness,^28,29^ the experimental results indicate that the device properties remain largely unaffected by the choice of substrate. The active layer of PDI-O dispersed in PS is responsible for the main emission characteristics of the laser, and since this material dominates the optical behavior of the devices, the substrate has a relatively small influence on emission properties. The resonant layer, made of DCG, also contributes significantly to the spectral characteristics, and its effect is independent of the substrate type. While FS and CA have different refractive indices and optical properties, these differences are not large enough to significantly affect the resonant modes in the DFB devices. The substrate primarily acts as a structural support and does not interfere substantially with light propagation in the active or resonant layers. This minimizes any significant impact that the substrate has on the overall optical properties the overall optical properties of the device.

### 2.3 Laser Wavelength Tuning through Mechanical Deformation (Bending)

Once it has been established that a DFB device can be fabricated on a flexible CA substrate with PDI-O films dispersed in PS, the next goal is to demonstrate that the emission wavelength of the device can be controlled through the mechanical deformation of the substrate, specifically by applying tangential bending to the device. This approach explores the ability to shift the emission wavelength over a range of approximately 10 nm by simply manipulating the bending angle. The bending induces changes in the geometry of the grating and, therefore, in the emission wavelength. The results obtained are discussed below, and an evaluation is made to determine whether the wavelength variation is primarily due to a change in the grating period or in the thickness of the device layers.

The effect of the DFB emission spectra upon bending can be seen in Figure 3. The DFB spectra for the TE_0_ mode of device 1/CA for different bending angles (positive or negative) are shown in Figures 3a and 3b. It is observed that, as deformation is applied to the device, its bending angle changes and the emission wavelength varies significantly. This change is continuous and covers a range of approximately 10 nm. To determine the values of the tuning angles θ, we took photographs of the devices after the application of the deformation, and then the images were analyzed using the ImageJ program.^30^ Angle measurements were taken multiple times to reduce errors (angle error ± 0.5°). The observed wavelength shift upon bending is a clear indication that the deformation of the device directly affects the optical structure of the grating, which in turn influences the emission wavelength according to Bragg’s diffraction principle. The wavelength shift occurs toward higher or lower wavelengths depending on the applied curvature. Figure 3c displays the variation of the DFB emission wavelength (left axis, in black) as a function of the device’s deformation angle. If it is assumed that the effective index of the structure is not affected by bending, according to eq 1, the change in wavelength would be due to a change in the grating period (ΔΛ). Such values are depicted on the right axis (in red) in Figure 3c. This change in the grating period would be explained by the compression or expansion of the grating grooves due to the bending of the device. A key question in this study is whether the assumption that the effective index of the structure (which is determined by the thickness of the active film, grating depth, and refractive index of the layers) is unaffected by bending is valid. Although deformation could theoretically induce changes in the thickness of the active or resonator layer, simulations using the software OMS^31^ showed that for thickness variation alone to explain the wavelength shift, the thickness would need to change by more than 100 nm (for the extreme cases studied, −10.1° and +11.7°). This suggests that the wavelength shift is not primarily due to a significant change in thickness but rather to variations in the grating period. In fact, these flexible DFB devices are designed in such a way that the layer thickness remains unaffected by deformation, allowing the device to maintain its structural integrity while adjusting the emission wavelength.

**Figure 3:**
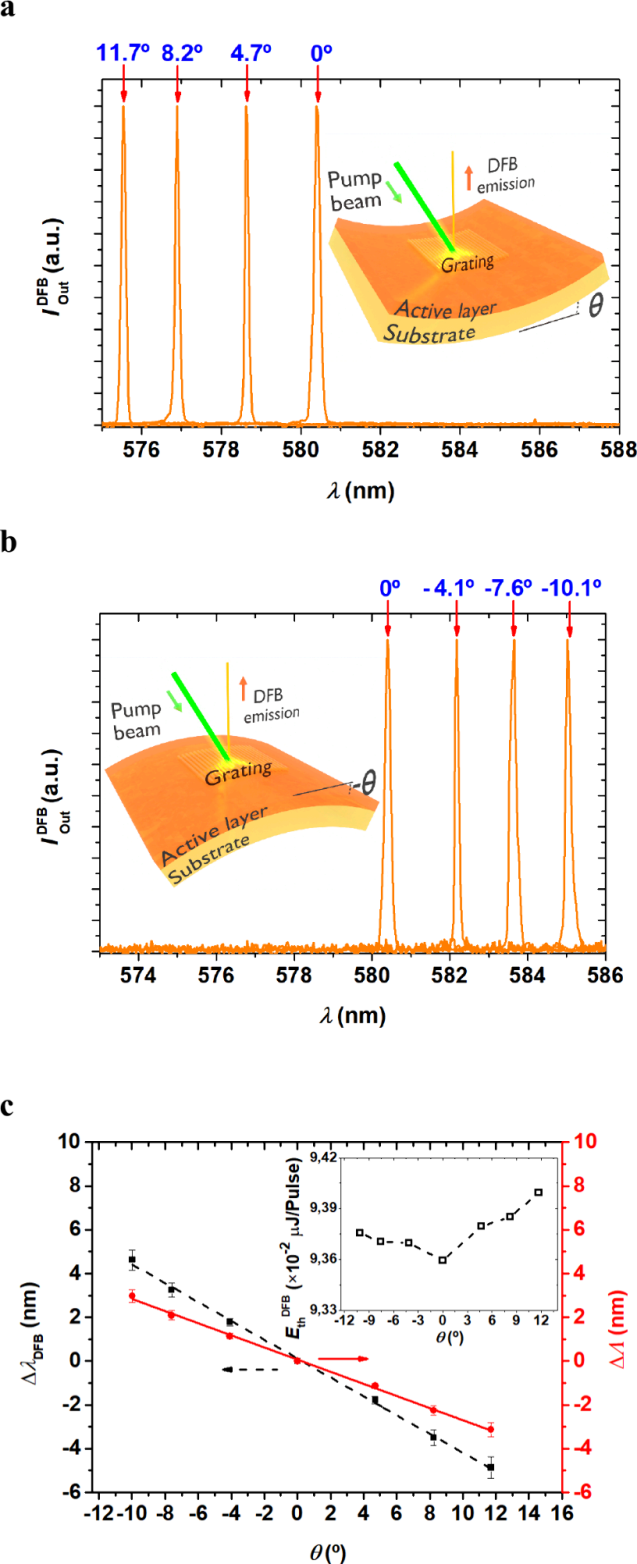
DFB laser spectra for the TE_0_ mode of device 1/CA for different bending angles θ: (a) positive angles (convex) and (b) negative angles (concave). (c) Variation of the DFB laser emission wavelength (left *y*-axis, in black) and the period variation of the films (right *y*-axis, in red) as a function of the deformation or bending angle of the devices. Angle error ± 0.5°.

While in this work the tunability of the DFB emission is achieved through substrate bending, an alternative strategy reported in the literature involves tuning by uniaxial stretching of flexible substrates. In such cases, the applied strain modifies both the grating period and the optical thickness of the waveguide layers, enabling substantial wavelength shifts (up to 10 nm or even 40 nm), as demonstrated with polydimethylsiloxane (PDMS) substrates.^21,22^ However, this approach requires materials with high elastic deformation capabilities. In contrast, the CA substrates used in the present study, exhibit limited elongation at break (∼5% under standard conditions),^32^ which restricts its practical use in stretch-tunable devices. This is consistent with other studies using CA or similar materials, where tuning is also achieved via bending.^20,23^ Nevertheless, CA might offer advantages over PDMS in terms of optical quality and mechanical stability. In preliminary experiments (unpublished), we attempted to fabricate DFB devices using PDMS as the substrate, but encountered significant difficulties in obtaining high-quality gratings due to the low mechanical rigidity of the material. In the few devices that were successfully fabricated, we observed high waveguide losses, elevated lasing thresholds, and poor photostability. While further systematic studies would be required to draw definitive conclusions in this regard, these observations highlight the importance of selecting an appropriate substrate depending on the desired tuning mechanism and device performance.

### 2.4 Refractive Index Sensing Applications

In this section we show results indicating the ability of DCG top-layer resonator DFB lasers with CA substrates to operate as refractive index sensors (see Figure 4). Particularly, the effect on the laser spectra of device 1/CA for both, TE_0_ and TM_0_, upon deposition of glycerine (G_100_) on top of the device, with respect to emission in air (A), is shown for device 1/CA in Figure 4a. The operation principle in these devices relies on a change in the emission laser wavelength, Δλ, due to a change in the effective index of the structure (see eq 1), as a consequence of a change in the refractive index *n* of the superstrate (a liquid instead of air). The wavelength shift obtained for the TM_0_ mode (Δλ_TM_ = 2.5 nm) is larger than that of the TE_0_ mode (Δλ_TE_ = 1.6 nm), in agreement with results reported previously for devices based on FS substrates.^24^

**Figure 4:**
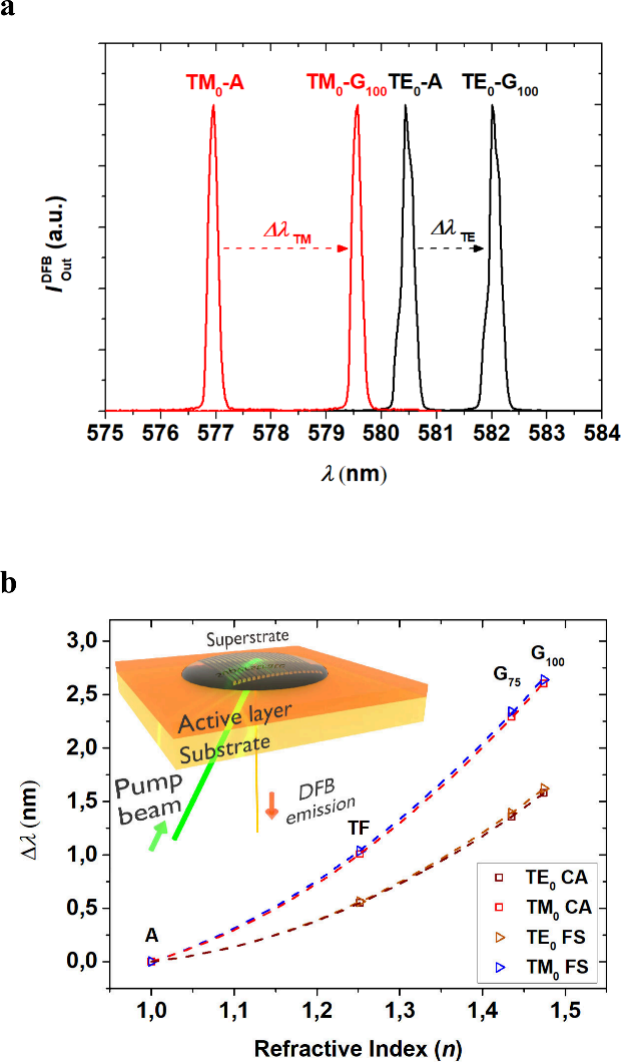
Properties of DFB laser sensors operating in TE_0_ and TM_0_ mode. (a) Laser spectra for device 1/CA upon exposure to pure glycerine (G_100_) and air (A). Each spectrum from the DFB sensor shows two peaks, corresponding to the TE_0_ (black line) and TM_0_ (red line) waveguide modes. (b) The laser wavelength shift (Δλ) from emission in air to emission with a given liquid superstrate, as a function of the superstrate’s refractive index (*n*) for devices 1/CA and 1/FS. The symbols correspond to data for superstrates A, TF, G_75_, and G_100_ (A: air; TF: tetradecafluorohexane; G_75_: glycerine solution at 75% in water; G_100_: glycerine 100%).

A more detailed analysis of the sensor performance was carried out by exposing the laser device to two additional liquids: Tetradecafluorohexane (TF) and a solution of glycerine at 75% in water (G_75_). To obtain the sensor *Sb*, the wavelength laser shift (Δλ) from emission in air to emission with a liquid superstrate was represented as a function of the superstrate refractive index *n* for devices 1/CA and 1/FS (see Figure 4b). The bulk sensor *Sb* is determined as the derivative of Δλ with respect to *n.* Numerical *Sb* values in the biological range (at *n* = 1.33) are similar for devices 1/CA and 1/FS, with larger values for the TM_0_ mode (*Sb* ^FS^ = 6.9 nm RIU^–1^, *Sb* ^CA^ = 6.8 nm RIU^–1^) than for the TE_0_ mode (*Sb* ^FS^ = 4.3 nm RIU^–1^, *Sb* ^CA^ = 4.3 nm RIU^–1^). It should be noted that the purpose here was simply to evaluate the effect of the substrate and not to optimize sensitivity. Further studies toward this end would imply the use of thinner active films, since it is known that this enables significant sensitivity improvements.^24^

## 3 Conclusion

The results obtained in this study demonstrate that top-layer DCG resonator DFB devices fabricated on flexible CA substrates exhibit excellent laser performance, comparable to that of equivalent devices using FS substrates. Notably, the use of flexible substrates enables precise and reversible tuning of the emission wavelength through mechanical deformation, with observed shifts of up to ∼10 nm. This level of tunability, although moderate, is consistent with values reported for other mechanically tunable DFB systems and is highly relevant for compact and low-power applications in sensing, on-chip spectroscopy, and wearable photonics.

The ability to tune the emission wavelength without requiring thermal or electrical modulation mechanisms represents a significant advantage in terms of integration and simplicity. Furthermore, the mechanical tuning strategy demonstrated here could be extended by combining it with previously reported approaches, such as the fabrication of multiple gratings with different periods on a single device.^11^ This would enable discrete or continuous wavelength selection over a broader spectral range, increasing the functional versatility of the platform.

The use of plastic materials such as CA, together with simple and cost-effective fabrication processes, also makes these devices an attractive option for scalable production. This is particularly advantageous for the widespread adoption of flexible optical systems in low-cost, portable, and adaptive applications, where tunability and mechanical robustness are essential.

Wavelength tuning in the plastic DFB devices is primarily governed by changes in the grating period induced by substrate bending, while the layer thickness remains nearly constant. This approach represents a promising route toward the development of flexible, tunable, and low-cost laser sources, with broad potential for future integration in adaptable photonic technologies.

## 4 Experimental Section/Methods

### Thin Film and Resonator Fabrication

Thin films of PS (Sigma-Aldrich, Mw = 35,000 g mol^–1^) doped with 1 wt % of PDI-O (Phiton) were prepared by spin coating at 3000 rpm on FS and CA substrates (25 × 25 × 1 mm^3^), using toluene as the solvent. Before deposition, the substrates were cleaned by ultrasound in alcohol for 5 min to remove any potential contaminants and ensure optimal adhesion of the material. The chosen concentration of the dye aimed to simultaneously optimize both the ASE threshold and the photostability of the films.^33^ The thickness of the obtained films was determined from the transmission spectrum analysis in the transparent region using a modification of the envelope method.^34^ Additionally, the percentage of PS in the solvent was carefully adjusted to achieve the appropriate thickness (*h*) for the films.

The first step in the resonator fabrication involved the deposition of a DCG layer by spin coating from an aqueous solution. The concentrations of inert gelatin (Russelot, 200 bloom) and ammonium dichromate, used as the sensitizer, were 2.2 wt % (with respect to water) and 35 wt % (with respect to gelatin), respectively. After the DCG film deposition, 1D gratings were recorded using HL with the Lloyd configuration,^11^ employing light from a solid-state laser emitting at λ_HL_ = 460 nm, with an average exposure of 800 μJ cm^–2^. The purpose of this high exposure was to harden the grating to enhance its stability and durability. During this step, the angle between the two interfering beams (ϕ) was chosen to obtain the desired grating period using the expression:

2 $$\Lambda =\lambda_{HL}/\left[2\;\sin\left(\text{ϕ}/2\right)\right]$$

In the case of CA substrates, the grating area was limited to 1 × 1 cm^2^ by placing a mask during exposure, since the substrate itself measures 2.5 × 2.5 cm^2^. This masking ensured that only the central region was patterned with the grating, minimizing edge effects and preventing deformation of the flexible substrate during exposure, which could otherwise lead to unwanted curvature or nonuniformity in the recorded grating. In contrast, for FS substrates, the full area of 2.5 × 2.5 cm^2^ was exposed and used for grating fabrication. Afterward, the DCG layers were desensitized in a cold-water bath (12 °C) to remove any residual effects of the sensitization. Finally, surface-relief gratings were obtained through a dry development process using oxygen plasma with a Diener Zepto surface treatment machine.^9,11^ All fabricated resonators had a grating depth of (110 ± 5) nm and were located on top of the active films (top-layer resonator geometry), enabling the construction of DFB laser devices with optimal characteristics. The indicated grating depth error of ± 5 nm corresponds to statistics across samples fabricated under identical conditions and is mainly due to slight differences in the thickness of the spin-coated DCG layers (before grating fabrication). This variation leads to minor shifts (±0.5 nm) in the DFB resonance wavelength due to changes in the effective refractive index. Note that the error reported in Table 2 for the DFB peak position corresponds to the spectrometer resolution for a single measurement.

Grating period reproducibility was assessed via diffraction efficiency measurements, by comparing the first-order diffraction angle after fabrication with the exposure angle used during holography. The statistical error in the grating period across samples fabricated under identical conditions was approximately 0.5 nm (shown in Table 2).

### Optical Characterization

The ASE and DFB laser measurements were conducted using as excitation source a pulsed Nd:YAG laser (10 ns, 10 Hz repetition rate), model Indi-40, provided by Spectra-Physics (MS Instruments Inc., Andover, MA, USA), operating at 532 nm. The power of the incident beam was modified using neutral density filters. For the ASE characterization, the beam was shaped into a stripe (0.5 × 3.5 mm^2^) using a cylindrical lens and an adjustable slit. This stripe was projected perpendicularly onto the film surface, with one of its edges aligned with the edge of the sample. The emitted PL was collected from this edge using a USB2000-UV–vis fiber spectrometer (Ocean Insight, Orlando, FL, USA), offering a nominal spectral resolution of 0.8 nm. For the DFB emission characterization, the pump beam incident on the sample (elliptical shape with a minor axis of 1.1 mm) was directed at an angle of approximately 30° relative to the normal of the sample plane. This slight deviation from normal incidence aimed to facilitate light collection, using an Ocean Optics MAYA2000 fiber spectrometer, in a direction perpendicular to the sample surface. The nominal wavelength resolution of this spectrometer is 0.07 nm. However, since the measured spectrum consists of discrete points separated by steps of 0.035 nm, the peak wavelength can be estimated with a resolution greater than that of the spectrometer by fitting the experimental data to a Lorentzian curve,^35^ or using a center of mass model approach.^36^ For the sensor measurements, the value of sensor resolution *r* is defined as twice the standard deviation of a set of successive measurements, and its magnitude is typically on the order of one picometer. The excitation and collection geometry (see Figure 4b) is carried out through the substrate, with the sample placed horizontally relative to the optical table to facilitate the deposition of liquid superstrates. This configuration, which requires a transparent substrate, prevents the pump beam from disturbing the analyte.

### Properties of Cellulose Acetate

The supplier of this material is Celanese-Clarifoil films, which provides A4 sheets of clear Clarifoil T24 in a thickness of 300 μm. This product is made up of several elements, with the most notable being CA at 78.39% of its composition, while 19.50% corresponds to a plasticizer. Additionally, it contains 0.25% of antiblocking and slip additives. Regarding its optical properties, the refractive index *n* of the material was measured by ellipsometry at the Institute of Ceramic and Glass (CSIC) in Madrid (see Figure S5 in the Supporting Information).

## Supplementary Material



## Notes

Effective refractive index as a function of film thickness, cutoff thickness values for the TE_0_ and TM_0_ modes, comparison of absorbances of FS and CA, SEM and FESEM images of DCG grating, DFB emission maps to assess spatial reproducibility across a sample, and refractive index as a function of wavelength for CA and FS (PDF)

## Abbreviations

A: air
a.u.: arbitrary units
ASE: amplified spontaneous emission
CA: cellulose acetate
DCG: dichromated gelatin
DFB: distributed feedback
FESEM: field emission scanning electron microscopy
FS: fused silica
FWHM: full width at half-maximum
G_100_: glycerin 100%
G_75_: solution of 75% glycerin in water
HL: holographic lithography
OMS: optical mode solver
PDI-O: perylene orange
PS: polystyrene
SEM: scanning electron microscopy
TE: transverse electric mode
TM: transverse magnetic mode
TF: tetradecafluorohexane
