# Supplementary material for: All-Plastic Organic Lasers with Top-Layer Polymeric Resonators: Tunable Emission through Bending and Application to Refractive Index Sensing
Source: ACS Appl Electron Mater. 2025 Oct 10;7(20):9480–8. doi: 10.1021/acsaelm.5c01592 (PMC12574577; doi:10.1021/acsaelm.5c01592)
Supplement: Supplementary file 1 [file el5c01592_si_001.pdf]

Supporting Information

All-plastic organic lasers with top-layer polymeric resonators: tunable emission through bending and application to refractive index sensing

Pablo Pasqués-Gramage,[1] Gema Calvillo-Solís, [1] Pedro G. Boj, [2] José A. Quintana, [2] José M. Villalvilla [1] and María A. Díaz-García [,1]*

[1]Dpto. Física Aplicada and Instituto Universitario de Materiales de Alicante (IUMA), Universidad de Alicante, Alicante, 03080 Spain. E-mail: maria.diaz@ua.es

[2]Dpto. Óptica, Farmacología y Anatomía and IUMA, Universidad de Alicante, Alicante, 03080 Spain.

Figure S1. Effective refractive index as a function of film thickness S3

Table S1. Cut-off values for the TE_0 and TM_0 modes S3

Figure S2. SEM and FESEM images of a DCG grating S4

Figure S3. DFB emission maps to assess spatial reproducibility across a sample................S5

Figure S4. Comparison of the absorbances of FS and CA ..…………………….……………. S6

Figure S5. Refractive index as a function of wavelength for CA and FS ……………………. S6

Dispersion equations for CA, FS and PS……………………………………………………S7

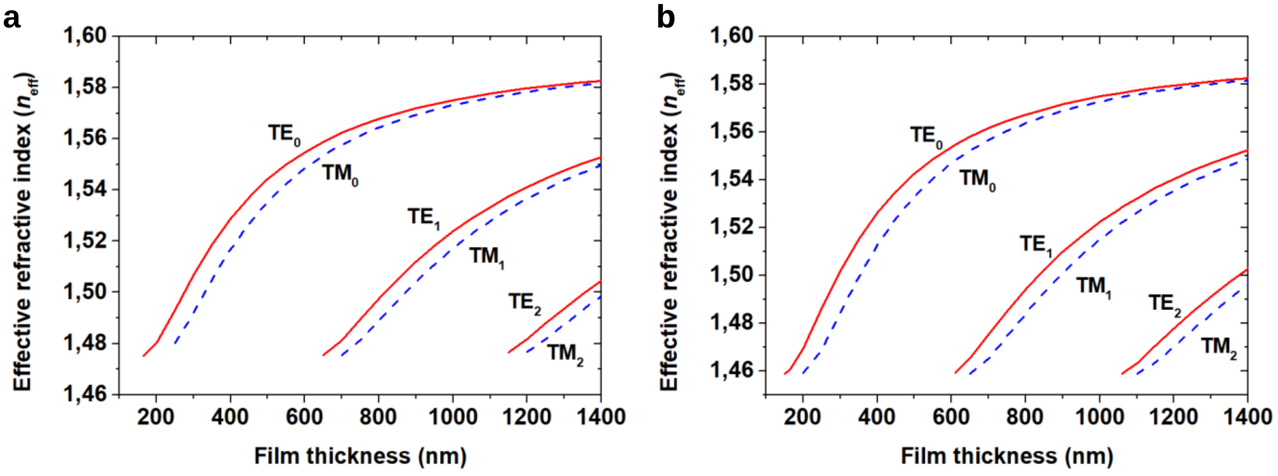

Figure S1. Effective refractive index (n_{eff}) for TE and TM modes as a function of film thickness for PS films doped with PDI-O deposited on a) CA substrate and b) FS substrate. The lines represent calculations based on the wave propagation equation at a wavelength of λ = 579 nm (solid lines for TE modes and dashed lines for TM modes, respectively).

Table S1. Cut-off values for the TE_0 and TM_0 waveguide modes propagating at λ = 579 nm along a thin PS film doped with PDI-O deposited on FS and CA substrates.

Substrate	$h_{cut\text{-}off}$ TE_0 (nm)	$h_{cut\text{-}off}$ TM_0 (nm)
CA	165	220
FS	150	200

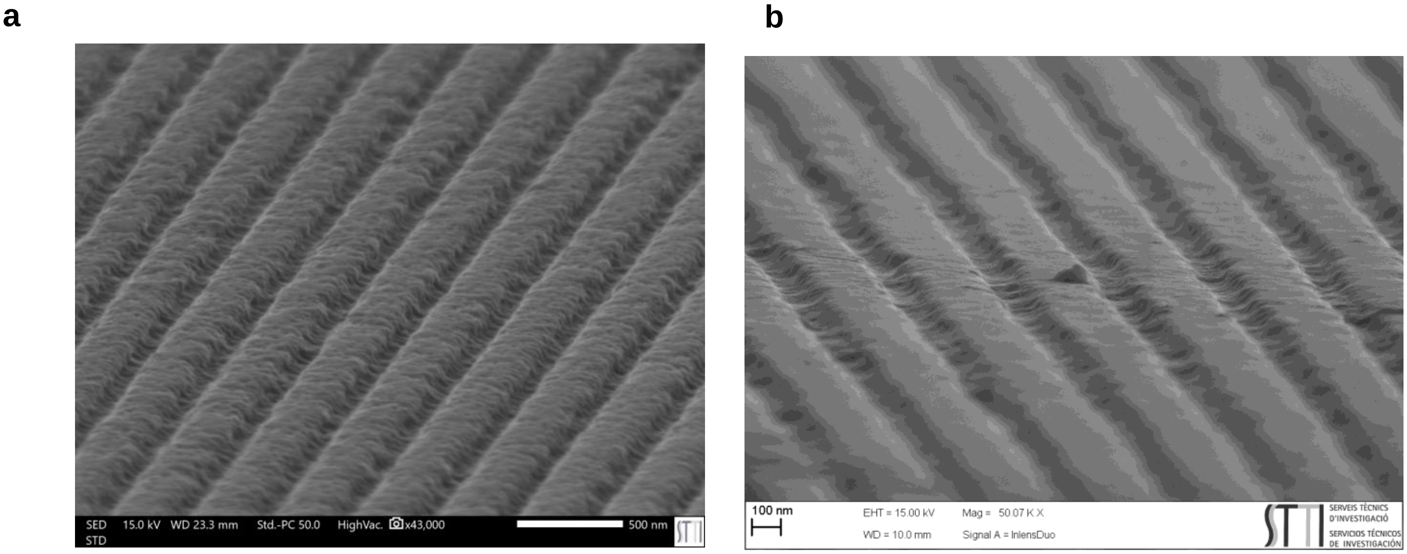

Figure S2. Top-view a) SEM b) FESEM images of a DCG grating with a period of Λ = 371.7 nm and a depth of about $d \approx$ 110 nm.

a

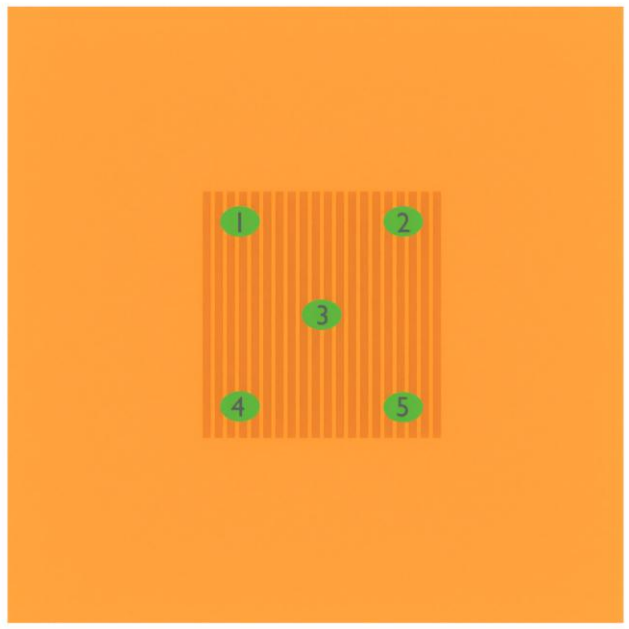

b

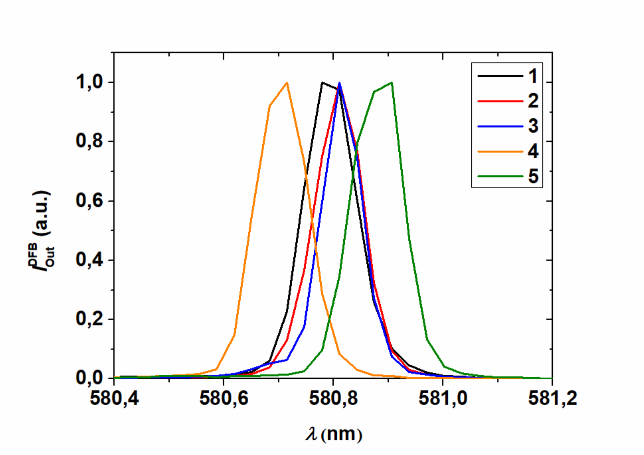

Figure S3. DFB emission maps to assess spatial reproducibility across a sample. (a) Scheme of a top view of the sample with the grating in the central region, highlighting five numbered zones (1–5) at which the excitation beam is focused to obtain the spectra. (b) DFB laser obtained from each of these zones.

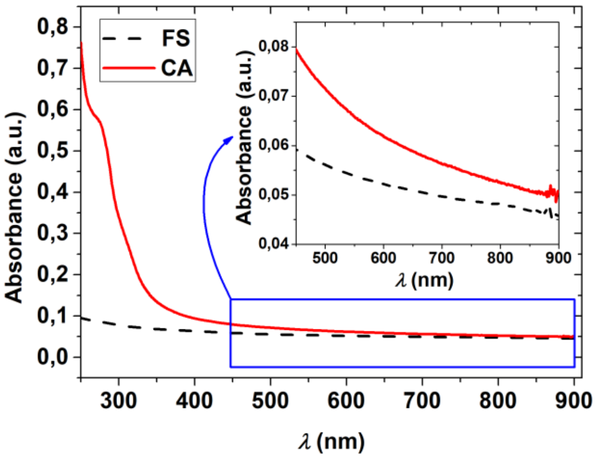

Figure S4. Comparison of the absorbances of FS and CA. In the top right corner, an enlarged detail of the absorbance curve of both substrates is shown in the range from λ = 450 nm to λ = 910 nm.

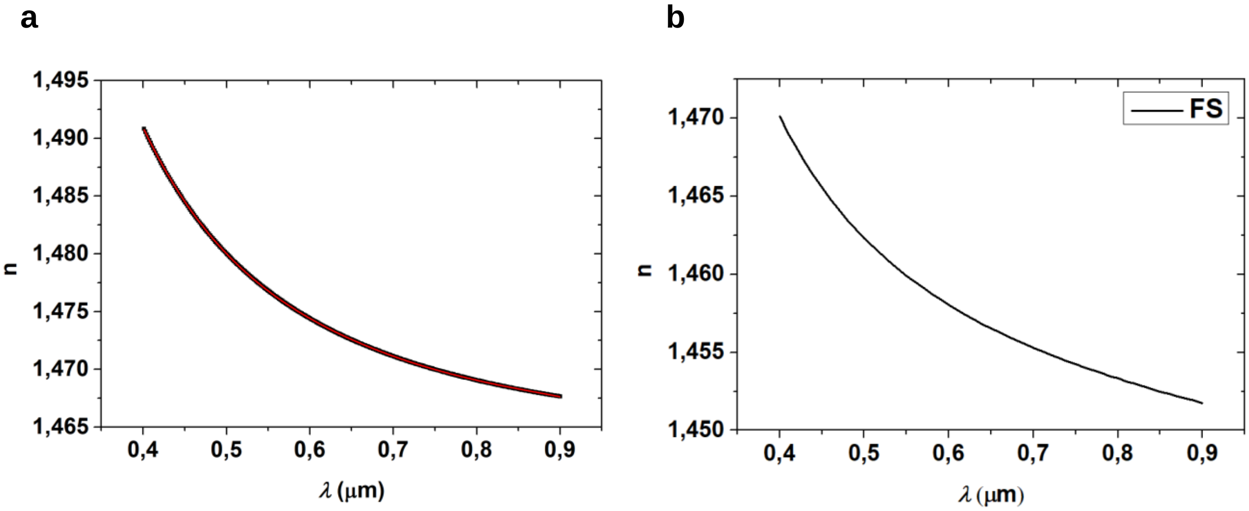

Figure S5. Refractive index as a function of wavelength for: (a) CA, measured by ellipsometry (black points) along with the corresponding fit (red line) from which the coefficients of the dispersion equation (S1) where obtained; (b) FS, calculated with dispersion equation (S2).

Dispersion equation for CA (coefficients obtained through the fit of experimental data, Figure S4a):

$$n^2 - 1.45643 = \frac{0.68238\lambda^2}{\lambda^2 - 0.01761} \quad \text{(S1)}$$

Dispersion equation for FS (taken from ref. [27] of the main manuscript):

$$n^2 - 1 = \frac{0.6961663\lambda^2}{\lambda^2 - 0.0684043^2} + \frac{0.4079426\lambda^2}{\lambda^2 - 0.1162414^2} + \frac{0.8974794\lambda^2}{\lambda^2 - 9.896161^2} \quad \text{(S2)}$$

Dispersion equation for PS (taken from ref. [27] of the main manuscript):

$$n^2 - 1 = \frac{1.4435\lambda^2}{\lambda^2 - 0.020216^2} \quad \text{(S3)}$$
